# Geometric image-based phenotyping and physiological analysis for validation of rice salinity tolerance screening under artificial pot conditions

**DOI:** 10.1186/s12870-026-08810-5

**Published:** 2026-04-23

**Authors:** Muhammad Fuad Anshori, Bambang Sapta Purwoko, Iswari Saraswati Dewi, Willy Bayuardi Suwarno, Sintho Wahyuning Ardie, Nono Carsono, Abd Haris Bahrun, Suharman Hamzah, Achmad Kautsar Baharuddin, Purnama Isti Khaerani, Bernadetta Rina Hastilestari, Zeeshan Ali, Majed Alotaibi, Nawab Ali, Mahmoud F. Seleiman

**Affiliations:** 1 Department of Agronomy, Faculty of Agriculture, Hasanuddin University, Makassar, 90245 Indonesia; 2 Rice Research Group, Faculty of Agriculture, Hasanuddin University, Makassar, 90245 Indonesia; 3 Department of Agronomy and Horticulture, Faculty of Agriculture, IPB University, Bogor, 16680 Indonesia; 4 Research Organization for Agriculture and Food, National Research and Innovation Agency, Cibinong, 16911 Indonesia; 5 Department of Crop Science, Faculty of Agriculture, Universitas Padjadjaran, Bandung, 45363 Indonesia; 6 Department of Civil Engineering, Hasanuddin University, Makassar, 90245 Indonesia; 7 Research Organization for Life Science and Environment, National Research and Innovation Agency, Cibinong, 16911 Indonesia; 8 Department of Plant Breeding and Genetics, Faculty of Agriculture and Environment, The Islamia University of Bahawalpur, Bahawalpur, 63100 Pakistan; 9 Department of Plant Production, College of Food and Agriculture Sciences, King Saud University, P.O. Box 2460, Riyadh, 11451 Saudi Arabia; 10 Department of Biosystems and Agricultural Engineering (BAE), College of Agriculture and Natural Resources, Michigan State University, East Lansing, MI 48824 USA

**Keywords:** Abiotic stress, Salinity stress screening, Image processing, *Oryza sativa*, Salinity soil screening

## Abstract

Salinity stress in coastal areas threatens the stability of rice production in Indonesia, necessitating innovative breeding strategies to adapt to this stress. In breeding, screening methods are crucial to improve selection effectiveness. One approach is pot selection on saline soil. However, this concept requires a precise approach, so integrating image-based phenotyping (IBP) screening and validation by physiological traits provides a rapid and effective approach to assessing salinity tolerance in rice genotypes. This study aimed to identify robust IBP traits for pot salinity screening and validate them through physiological response patterns among rice genotypes under salinity conditions. Six rice genotypes were evaluated under normal and saline environments using artificial pot trials. IBP traits related to plant geometry were quantified and complemented with physiological indicators, including Na⁺/K⁺ balance, chlorophyll pigments, and proline accumulation. Based on the result, perimeter and ferret were identified as effective IBP selection criteria. Both criteria captured differences in osmotic regulation and photosynthetic performance under salinity stress. Principal component analysis clearly separated tolerant, moderately tolerant, and sensitive genotypes, with geometric traits contributing most strongly to genotype discrimination. It supported a bit of physiological responses, which revealed distinct tolerance patterns. Tolerant genotypes (Pokkali, HS4.15.1.70, and HS4.15.2.4) maintained better Na⁺/K⁺ balance, lower chlorophyll loss, and adaptive proline responses, while sensitive genotypes (IR 29 and Ciherang) showed pronounced ionic imbalance and chlorophyll reduction; HS4.45.1.66 exhibited intermediate responses. The integration of IBP and physiological traits offers a practical framework for high-throughput salinity screening.

**Supplementary Information:**

The online version contains supplementary material available at 10.1186/s12870-026-08810-5.

## Introduction

Ensuring consistent rice development is crucial for maintaining food production stability in several Asian countries [1, 2]. As a staple food, rice significantly influences inflation in these regions, necessitating a balance between production and consumption in Asian nations, including Indonesia [3, 4]. Although rice production has seen recent increases, future growth may be hindered by climate change and global warming [4–6]. Furthermore, phenomena such as El Niño and La Niña can lead to crop failures during specific periods [7, 8]. As an archipelagic nation, Indonesia is particularly susceptible to the various impacts of global warming and climate change [9, 10]. This susceptibility may result in rising sea levels, leading to seawater intrusion and increased salinity in coastal regions [11, 12]. Consequently, rice production in Indonesia, especially in coastal areas, is likely to be affected. Salinity tolerance in rice is governed by physiological mechanisms, including ion selectivity (Na⁺ exclusion and K⁺ retention) and osmotic stability, which influence water status, leaf expansion, and photosynthetic capacity under saline conditions. These processes affect plant growth and architecture at the whole-plant level [13].

Rice cultivation in saline regions can be enhanced using intensification strategies [14–16]. A key strategy is genetic improvement via plant breeding, which enables adaptation to coastal and salinity-affected environments [17–19]. The process of breeding rice for salinity stress tolerance has been documented. However, developing salinity-tolerant varieties depends on screening methodologies used to assess stress tolerance. As highlighted by [20–22], multiple selection techniques exist for breeding salinity-tolerant rice varieties in the Philippines. Direct selection of saline land, involving screening within target environments, is one technique [23]. Despite its potential, this approach is costly and complex, especially during initial breeding with large populations [24]. Therefore, artificial screening methods are advantageous in early breeding stages with extensive populations, as indicated in previous studies [25].

**Fig. 1:**
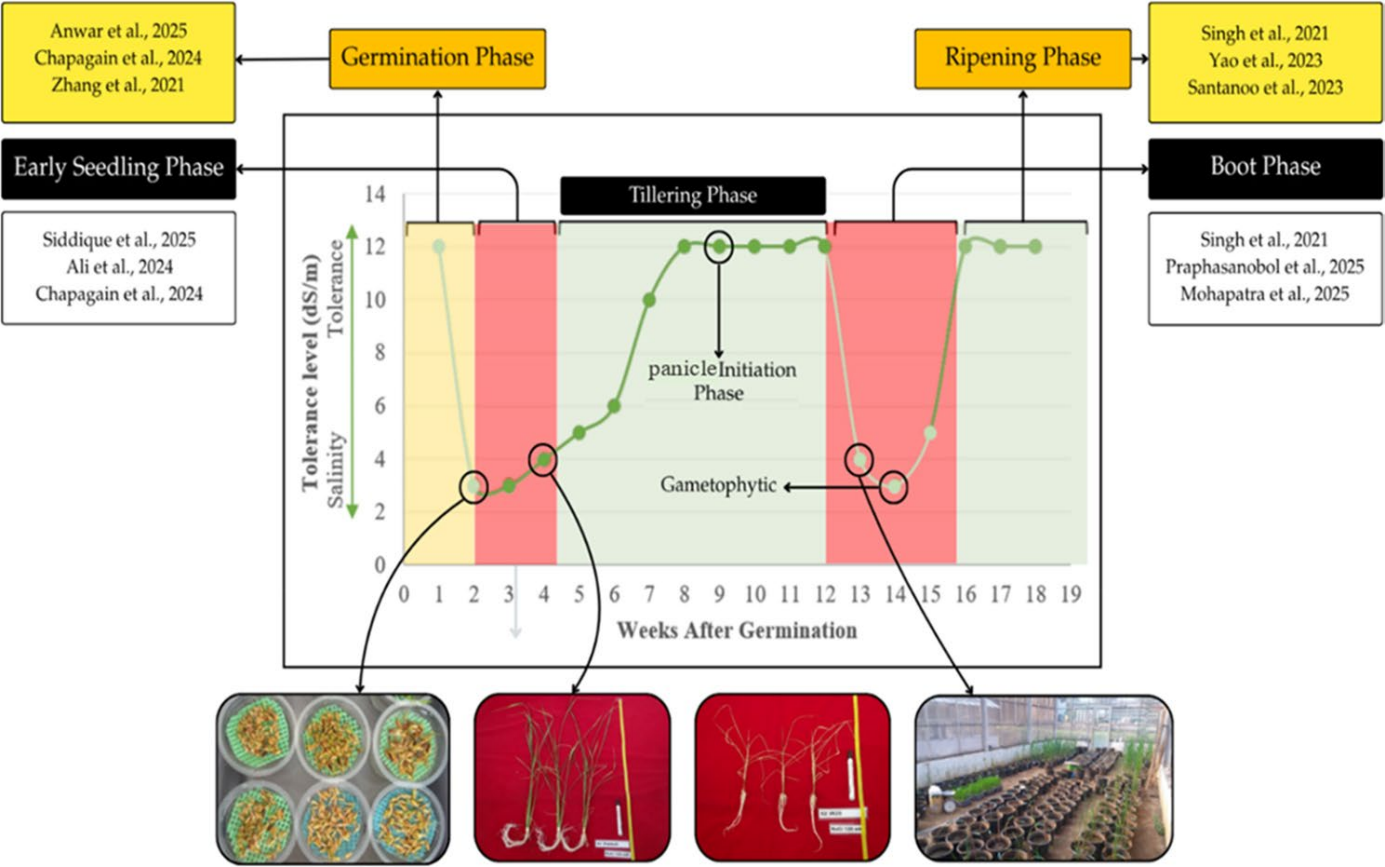
Dynamic of salt tolerance across growth phase in rice. Note: Adapted from Singh et al. [26]

The integration of artificial techniques for salinity screening is aligned with the critical stages of rice development under stress conditions (Fig. 1) [26–28]. As highlighted by [29–31], salinity stress screening in rice encompasses three primary stages: germination, seedling, and reproductive stages. Germination screening is especially advantageous because it is performed in the initial stages of plant growth [32]. However, this stage is less effective for screening because it relies on high concentrations and osmotic selection potential [33]. At this stage, NaCl toxicity is not strongly expressed because rice plants have not yet formed their leaves. Consequently, the seedling-stage stress approach is commonly used for the rapid screening of rice genotypes for salinity tolerance [34]. Numerous studies underscore their efficacy, as the effects of salinity are more pronounced during this stage. Nonetheless [22], proposed that tolerance to salinity stress during the vegetative stage may not correspond with tolerance in the reproductive phase, due to the cumulative nature of NaCl toxicity, which becomes apparent in the reproductive phase [35]. Rice salinity tolerance varies across growth stages, including germination [32, 36, 37], early seedling [36, 38, 39], PI (panicle initiation), booting [26, 40, 41], and ripening [26, 35, 42], as shown in Fig. 1. This variability indicates that effective screening should account for cumulative stress effects, making artificial pot-based selection particularly suitable for field evaluation under saline conditions (Table 1). Pot-based screening allows for precise and reproducible control of salinity stress, enabling accurate genotype comparisons. Although these controlled conditions may not fully capture the complexity of the field, they are highly appropriate for early-stage screening and trait characterization prior to large-scale field validation. Despite its advantages, salinity evaluation in artificial pots often relies on visual scoring and subjective assessment of plant vigor [43], which may lead to inconsistent or inaccurate classification of genotype tolerance. Moreover, salinity tolerance in double haploid (DH) rice genotypes has not been extensively evaluated using objective phenotyping approaches. Therefore, a precise approach is essential for evaluating rice tolerance using the artificial pot screening method. One viable approach is image-based phenotyping (IBP).

**Table 1:** Overview and research gaps of salinity tolerance screening approaches in rice (2014–2025)

Reference	Country	Plant Materials and Population	Screening Method	Major Finding	Limitation
[44]	Ethiopia	15 genotypes (IRRI lines)	EC 0, 4, 8, 12 dS/m; 5 kg soil/pot; factorial CRD	IR66946-3R-176-1-1 and IR68144-2B-2-2-3-2 were tolerant across stages	No physiological traits; focused only on yield
[45]	USA	74 genotypes	Pure sand medium; EC 6, 12 dS/m; movable shelter; SSRI, PCA	Root traits (volume, length) best descriptors; FED473 most tolerant	No reproductive stage data; no canopy imaging
[46]	Indonesia	Pokkali & IR29	Soil-based pot trial; EC 2.14–8.43 dS/m; RCBD	Total tiller number best selection trait; EC 5.62 dS/m optimal	Only 2 genotypes; no physiological or image traits
[47]	Indonesia	42 genotypes (36 DH lines + 6 checks)	EC ~ 5.6–5.8 dS/m; pot trial to reproductive stage; STI, path analysis	Productive tillers and filled grains best predictors of yield	No image-based phenotyping; limited physiological markers
[30]	Thailand	10 selected varieties from 382	Pot trials at seedling, tillering, flowering; EC 6–16 dS/m	LLR054, LLR365, LLR216 tolerant across stages; LLR050, LLR449 high yield	No physiological markers; image traits not assessed
[48]	Thailand	6 varieties	Pot trials at reproductive stage; EC 0.1–0.4% NaCl (~ 8 dS/m)	SPAD, Na^+^/K^+^, panicle traits correlated with field performance	No image-based phenotyping; small genotype set
[34]	Philippines	8 cultivars	Soil-filled trays (30 L); EC 10 dS/m; drydown to 30% FC	Standardized drought+salinity protocol; chlorophyll fluorescence correlated with yield	Focused on seedling stage; no reproductive data
[49]	Australia	2 rice genotypes	Pot trial, RGB imaging, PCA, EC 12 dS/m	Perimeter and compactness were key indicators of salinity response	Limited physiological validation
[50]	China	40 genotypes	Pot trial, EC 10 dS/m, ion content, growth rate	K⁺/Na⁺ ratio and proline were strong tolerance markers	No image-based phenotyping was used
**Current** **Research**	**Indonesia**	**Pokkali, IR29, moderate genotypes**	**Artificial pot trials; EC 6–8 dS/m**	**Image-based traits combined with K/Na, proline, and chlorophyll to distinguish tolerance classes**	**Novel integration fills the gap in image-physiology linkage**

Bold text indicates the present study and its contribution relative to previous studies

Image-based phenotyping (IBP) can support the objective, high-throughput selection of salinity-tolerant genotypes in rice breeding programs by translating physiological tolerance mechanisms into measurable morphometric traits. IBP analyzes plant phenotypes through image analysis, as detailed by [31,51], and [52]. The IBP framework evaluates plants by analyzing morphological dot pixels in images, offering a more comprehensive assessment than traditional evaluations [53, 54]. This concept has been discussed by [31] for evaluating abiotic tolerance, including salt tolerance in rice [25, 55]. Salt tolerance is a quantitative trait (QTL), with quantitative trait loci identified under stress conditions [26, 56, 57], necessitating a holistic plant assessment. Scoring-based evaluations are less aligned with genetic potential, making image-based assessments that correlate plant growth responses to environmental factors more effective for artificial screening in pots [45, 9]. Several IBP characteristics can serve as evaluation criteria for evaluating salt-tolerant genotypes. The geometric concept within IBP focuses on the area and shape of captured plant images [51, 58], differing from the colorimetric concept, which emphasizes color reflection [25, 59]. The geometric concept is suitable for assessing the plant growth ratio in response to salinity stress up to the reproductive phase [49, 51], while the colorimetric concept is used for rapid screening during the seedling phase [25, 31, 60], making the geometric approach more optimal [51, 61]. In addition, image-derived geometric traits represent phenotypic responses to salinity stress and provide quantifiable indicators for plant breeding selection. These traits capture whole-plant responses related to ion homeostasis and osmotic stability, offering practical targets for identifying salinity-tolerant genotypes. This study hypothesizes that rice genotypes that maintain ion balance under salinity stress will show stable geometric traits through image-based phenotyping, whereas sensitive genotypes will exhibit morphological reductions. However, these geometric concept evaluations require further exploration of tolerance, including physiological potential [25]. Salinity stress induces osmotic, toxicity, ion homeostasis, and free radical stress, each with a distinct tolerance mechanism [33, 62, 63]. A physiological approach is essential for determining the response and tolerance mechanisms [15, 21]. Integrating IBP with physiological approaches is crucial for optimizing methods and mapping the tolerance response patterns of rice genotypes in artificial salinity screening. This study aimed to (1) identify effective IBP criteria for optimizing artificial salinity screening in pots and (2) validate them through physiological response patterns among double haploid rice genotypes under salinity conditions and comparison with mini literature review.

## Materials and methods

### Research design

This study was conducted at the Cimanggu Experimental Garden in Bogor and the Faculty of Agriculture at Hasanuddin University. A nested randomized block design was employed, with replicates nested within two salinity screening environments: normal and saline environments. Six rice genotypes were utilized, specifically the varieties HS4.15.1.70 (DH breeding lines), HS4.15.2.4 (DH breeding lines), HS4.45.1.66 (DH breeding lines), Ciherang (Mega variety in Indonesia), Pokkali (IRRI’s tolerant check variety), and IR 29 (IRRI’s sensitive check variety). The selection of these varieties was based on previous studies [25, 54, 60], categorizing HS4.15.1.70, HS4.15.2.4, and Pokkali as tolerant, HS4.45.1.66 as moderate, and Ciherang and IR29 as sensitive. Each genotype was replicated thrice in each environment, resulting in 36 experimental units. Each unit comprised three samples, resulting in a total of 108 samples.

### Research procedure

This study followed methodologies in [46, 47], with adaptations specific to this research. Rice seeds (*Oryza sativa* L.) were sown in containers for 25 d until reaching the physiological seedling stage. The seedling media comprised sterile latosol soil and biochar rice husks with a volume ratio of 3:1. The planting pot medium (12 L volume) was prepared three days before transplantation, using sterile latosol soil, mature goat manure, and NaCl solution in a 7:1:3 ratio. NaCl was administered at transplantation and not added again until harvesting. The NaCl solution had an Electrical Conductivity (EC) level of 6 dS m⁻¹. This solution was mixed into the growing medium in 25 cm diameter pots and homogenized until achieving semi-saturated mud consistency. Seedlings underwent pre-acclimatization 20 days after sowing, with exposure to 3 dS m⁻¹ salinity solution for 48 h at room temperature to mitigate osmotic and transplant shocks [25, 46, 47]. After pre-acclimatization, two seedlings were transplanted per pot. Water was added every two days to maintain salinity stress. The EC of stagnant water was monitored using a Jenway model 470 EC meter before and after watering. Maintenance included weeding, fertilization, pest and disease management, and harvesting. Fertilization used 6 g/pot of NPK 15:15:15 pearl fertilizer one week after planting (WAP) and 1.5 g/pot of urea at 3 and 7 WAP for vegetative growth and panicle formation. Harvesting occurred when 80% of panicles turned yellow, indicating seed maturity.

### Data observation

**Fig. 2:**
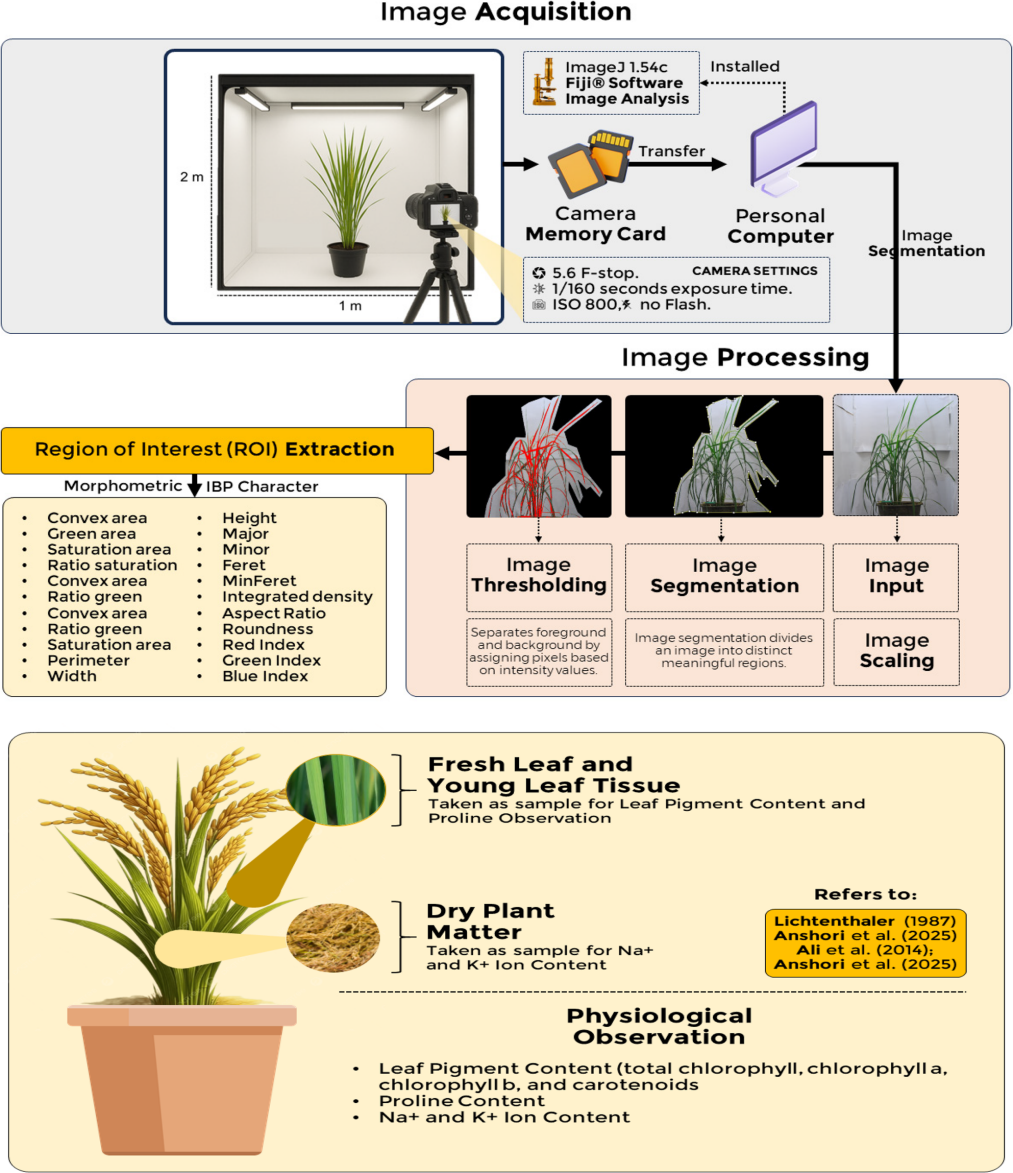
The IBP observation and analysis study framework

Data observation focused on geometric IBP characteristics and physiological characteristics, as shown in Fig. 2. IBP characteristics observations were adjusted according to [25, 31, 53, 55]. Plant images were captured using a Canon EOS 1200D RGB camera in a portable photo studio measuring 2 m × 1 m × 1 m. The studio had a white background and two 12-watt white LED lights for uniform lighting. Image acquisition used a top-mounted aperture above the studio for standardized front-view plant perspectives. Camera settings remained consistent: 5.6 F-stop, 1/160 seconds exposure time, ISO 800, and no flash to minimize glare and shadows [55]. Photographs were taken 60 days after planting. Phenotypic measurements from scans were obtained semi-automatically using Fiji^®^ software. 1.54c. Scaling factors were established using ruler marks for absolute morphometric measurements. In triangle mining, foreground objects were differentiated from background pixels. To enhance spectral differences between plant and non-plant characteristics, RGB color space was converted to Hue, Saturation, and Brightness (HSB) stack. S (0–100%) and B (0–100%) denote color shade and brightness, while H (0–360°) identifies pure color, making HSB effective for foreground selection and background-fingerprinting. The HSB histogram was computed, and optimal threshold (THR), defined as maximum distance between histogram peak and farthest line tip, was determined automatically. Pixels exceeding THR were classified as background (“0”), others as plant (“1”). An RGB image was superimposed on the binary mask to extract the region of interest (ROI). The ROI was analyzed to measure convex area, green area, saturation area, ratio of saturation and convex area, ratio of green and convex area, ratio of green and saturation area, perimeter, width, height, major, minor, Feret, integrated density, MinFeret, aspect ratio (AR), roundness, red index (RI), green index (GI), and blue index (BI).

Physiological observations are described in [25]. Parameters assessed included leaf pigment content (total chlorophyll, chlorophyll a, chlorophyll b, and carotenoids), proline, and Na + and K + ion contents. For chlorophyll quantification, fresh leaves from the oldest Sect.  (0.1 g from leaf center) were used. The leaves were homogenized, and 2 mL of 80% acetone was added to the mixture, then transferred to a falcon tube until reaching 10 mL. The mixture was centrifuged at 3,000 rpm for 10 min at 4 °C. Chlorophyll concentration in the supernatant was measured using a spectrophotometer at 470, 464, and 663 nm wavelengths. The concentrations of chlorophyll a, b, and carotenoids were calculated using the formula provided by Kiefer [64]. Proline analysis was conducted following procedures from [25] and [65]. Fresh young leaves (0.25 g) were collected and homogenized. Subsequently, 5 mL of 3% sulfosalicylic acid was added to each sample. The sample was centrifuged at 3600 rpm for 25 min at 4 °C. Then, 2 mL of supernatant was transferred to a vial and combined with 2 mL of ninhydrin dissolved in glacial acetic acid, 6 M orthophosphoric acid, and 2 mL of glacial acetic acid. The reaction mixture was incubated for 1 h at 80 °C and cooled to room temperature. Then, 4 mL of toluene was added and vortexed. The mixture stood for 5 min until a phase boundary formed. Proline in toluene was quantified using a spectrophotometer at 520 nm, and concentration was determined using a standard curve. Na + and K + ion contents were quantified for three genotypes representing distinct clusters: Pokkali (tolerant cluster), HS4.45.1.66 (moderate group), and IR 29 (sensitive group). These ions were analyzed using atomic absorption spectrophotometry at the Integrated Laboratory, Department of Agronomy and Horticulture, IPB University. Samples of 5 g dry matter from each genotype were analyzed.

### Data analysis

The data were systematically analyzed by integrating several concepts [25, 31, 47, 53, 55]. Analysis began with variance analysis as the initial phase in evaluating IBP characteristics as evaluation criteria. Characters identified in the Analysis of Variance (ANOVA) were analyzed using the Best Linear Unbiased Estimator (BLUE) and stress tolerance index (STI). BLUE analysis was conducted using CYMMIT’s META-R software, while STI analysis used Excel 365. The BLUE analysis mitigates random effects within a dataset, optimizing each genotype’s potential for a trait across environments. The tolerance potential was then optimized for interaction between environments using the STI concept. STI’s efficacy has been documented in studies, including rice tolerance to salinity stress. This concept enhances a genotype’s performance potential in two environments (saline and normal) relative to its potential under normal conditions, facilitating identification of dynamic adaptability to stress conditions. The BLUE and STI analyses were followed by multivariate analyses, specifically principal component analysis (PCA) and factor analysis. PCA was executed using RStudio 3.6.3 with the factoextra package, and factor analysis used Minitab v. 17 software. The application of these concepts has been reported by [66]. The two analyses offer distinct perspectives, though diversity partitioning in both is based on eigenvectors and eigenvalues. PCA aggregates and distributes data diversity into several dimensions while preserving original total diversity, providing objective mapping and avoiding multicollinearity. Factor analysis identifies dominant characteristic variables of a dimension partition by reducing diversity of characters with low covariance and enhancing diversity of characters with high covariance, making selection more objective. The combination facilitates comprehensive evaluation of criteria for genotype tolerance potential in soil salinity screening. Multivariate analysis results were validated through BLUE analysis and relative decrease and increase [25, 54]. The relative changes in physiological traits were followed by PCA analysis with selected IBP traits, validating relationships between IBP traits and physiology in objects with limited participants. Physiological analysis was deepened with comparative analysis of Na^+^ and K^+^ ion content against selected genotypes representing phenotypic tolerance classes. The distribution of Na^+^ and K^+^ contents was analyzed using RStudio (version 3.6.3) software.

## Results

### IBP trait variation and genotypic grouping under salinity stress

Variance analysis was performed using the log data transformation concept, as shown in Table 2. Based on this table, the characteristics of the green area, saturation-convex ratio, green-convex ratio, green-saturation ratio, Minor, Aspect ratio, roundness, red index, and green index still had a coefficient of variance above 20%. In addition, the variance in genotype and genotype-stress interactions showed no significant effect on the overall image-based phenotyping characteristics during screening for soil salinity in pots. Stress was the only source of diversity that affected most IBP characteristics, except for the green-convex ratio, green-saturation ratio, height, red index, green index, and blue index.

**Table 2:** ANOVA of image-based phenotyping (IBP) based on plant morphometrics

Characters	Stress	Genotype	GxS	CV_trans
Convex Area (conv)	0.002^**^	0.513	0.967	8.16
Green Area (green)	0.007^**^	0.707	0.997	23.59
Saturation Area (sat)	0.000^**^	0.975	0.576	12.69
Ratio of sat/conv	0.000^**^	0.903	0.449	36.66
green/conv	0.638	0.369	0.513	229.55
green/sat	0.173	0.635	0.581	137.65
Perimeter	0.015^*^	0.713	0.994	16.65
Width	0.000^**^	0.401	0.547	10.45
Height	0.109	0.448	0.506	5.79
Major	0.006^**^	0.838	0.628	9.19
Minor	0.001^**^	0.923	0.854	26.43
Feret	0.007^**^	0.173	0.411	4.78
integrated density	0.016^*^	0.892	0.909	12.85
Minimum Feret	0.000^**^	0.587	0.394	18.02
Aspect ratio	0.030^*^	0.589	0.973	56.35
Roundness	0.028^*^	0.591	0.982	26.83
Red Index	0.449	0.844	0.709	20.51
Green Index	0.562	0.924	0.878	27.17
Blue Index	0.062	0.933	0.279	15.14

Single asterisk (*) indicates significance at the 5% level (α = 0.05), while double asterisk (**) indicates significance at the 1% level (α = 0.01) based on the F-Test

**Fig. 3:**
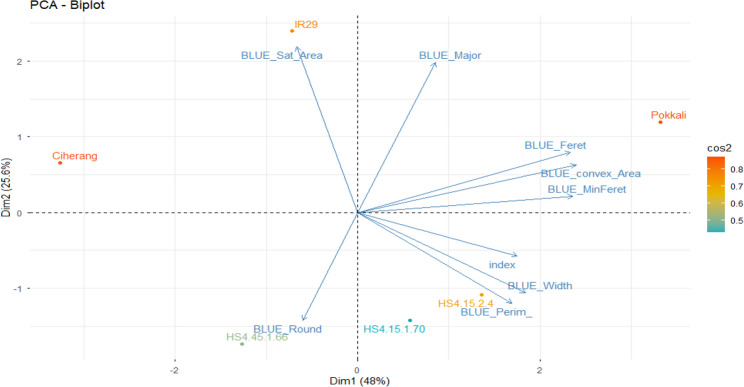
Biplot PCA analysis of STI IBP relationships between image-based geometric traits and rice genotypes under salinity stress

The IBP characteristics identified as significant in the ANOVA were further analysed independently using the BLUE method, as follows: All BLUE-IBP results are presented in Supplementary (1) The results of the BLUE analysis were subsequently refined using the stress tolerance index, as presented in Table 3. According to this table, the HS4.15.1.70 line exhibited the lowest STI potential for the IBP characteristics of the saturation area (0.15), major (0.58), and round (0.44). Conversely, the HS4.15.2.4 line demonstrated the highest potential for the perimeter characteristics (0.61). The HS4.45.1.66 line exhibited the highest STI potential for the round characteristic (1.37). The Ciherang variety displayed the highest STI potential in the saturation area (0.40), but exhibited the lowest STI potential in the convex (0.30) and saturation (0.27) areas, and for perimeter (0.37), width (0.57), and minferet (0.35). The IR29 variety demonstrated the highest potential for the major characteristics (0.94). Meanwhile, the Pokkali variety showed the greatest STI potential for the convex area (0.99), width (0.78), feret (1.13), and minferet (0.63) measurements. The PCA STI analysis revealed a substantial total dimension of PC1 (48%) and PC2 (25.6), with a cumulative total of 73.6% (Fig. 3). Based on the PCA grouping, the Pokkali variety was classified in quadrant 1, whereas the IR29 and Ciherang varieties were grouped in quadrant (2) The HS4.45.1.66 line was placed in quadrant 3, and HS4.15.2.4 and HS4.15.1.70 lines were categorized in quadrant 4. Additionally, based on the grouping of IBP characteristics, the Feret, Minferet, convex area, width, and Perim characteristics formed acute angles with the selection index.

**Table 3:** Stress tolerance index (STI) of selected IBP characters based on ANOVA

Genotype	Class	ConvexArea	Sat_Area	Perim	Width	Major	Feret	MinFeret	Round
HS4.15.1.70	Tolerance	0.53	0.15	0.55	0.59	0.58	0.84	0.56	0.44
HS4.15.2.4	Tolerance	0.63	0.20	**0.61**	0.66	0.71	0.88	0.61	0.77
HS4.45.1.66	Moderate	0.43	0.19	0.32	0.75	0.70	0.74	0.38	**1.37**
Ciherang	Sensitive	0.30	**0.40**	0.27	0.37	0.64	0.57	0.35	0.65
IR29	Sensitive	0.54	0.35	0.32	0.47	**0.94**	0.92	0.51	0.46
Pokkali	Tolerance	**0.99**	0.34	0.43	**0.78**	0.87	**1.13**	**0.63**	0.59
Mean		0.57	0.27	0.42	0.60	0.74	0.85	0.51	0.71

The bold and purple color indicated the highest and lowest

*Sat_Area* Saturation area, *Perim*  Perimeter, *MinFeret * Minimum of ferret, *round * Roundness

### Factor analysis of IBP traits and physiological validation under salinity stress

The results of the factor analysis of the IBP characters related to the selection index are listed in Table 4. The analysis revealed a total diversity of 99% across the entire dataset, which was categorized into three factor dimensions. Within the factor dimension 1, the ferret character exhibited the most significant diversity, with a value of 0.54. In factor dimension 2, the Perim character demonstrated dominant diversity with a value of -0.91. Meanwhile, in factor dimension 3, width emerged as the predominant IBP characteristic, with a value of -1.15.

**Table 4:** Factor analysis of IBP traits related to the selection index

Variable	Factor1	Factor2	Factor3	Communality
BLUE_convex_Area	0.49	0.22	0.01	0.98
BLUE_Perimeter	-0.35	**-0.91**	-0.04	1.00
BLUE_Width	-0.32	0.06	**-1.15**	1.00
BLUE_Feret	**0.54**	0.23	0.12	0.98
BLUE_Minimum Feret	0.30	-0.34	0.33	1.00
Variance	2.38	1.46	1.11	4.96
% Variance	0.48	0.29	0.22	0.99

Bold indicates the highest variance in each factor dimension

The physiological potentials of the six genotypes were first analyzed using the BLUE concept (Supplementary 2). The BLUE results were optimized into relative decrease and relative increase forms (Table 5). According to the data presented in this table, the Pokkali variety exhibited the smallest relative decrease in total chlorophyll (11.50%), chlorophyll a (11.60%), and chlorophyll b (11.24%). In contrast, the HS4.15.2.4 line demonstrated the most significant relative decrease (46.04%) and the least relative increase (-9.60%). The HS4.45.1.66 line also recorded the highest relative decrease in total chlorophyll (52.37%) and chlorophyll b content (55.71%), while also showing the most substantial relative increase of 1726.25%. The Ciherang variety experienced the greatest relative decrease in chlorophyll a (52.93%). Meanwhile, the IR29 variety exhibited the smallest relative decrease in carotenoids (-15.30%).

**Table 5:** Relative decrease and relative increase of physiological characteristics

Genotype	RD_Chl Tot	RD_Chl a	RD_Chl b	RD_Car	RI_proline
HS4.15.1.70	26.36	26.77	25.23	15.58	322.50
HS4.15.2.4	38.59	42.69	27.09	46.04	-9.60
HS4.45.1.66	52.37	50.90	55.71	31.01	1726.25
Ciherang	50.13	52.93	42.33	37.98	133.75
IR29	31.85	32.21	30.88	-15.30	1001.18
Pokkali	11.50	11.60	11.24	9.99	513.45

The blue and purple colors indicated the best and worst

*RD_Chl Tot* Relative decrease in total chlorophyll, *RD_Chl a* Relative decrease in chlorophyll a, *RD_Chl b *Relative decrease in chlorophyll b, *RD_Car *Rrelative decrease in carotenoid, *RI_proline* Relative increase in proline

### Correlation and PCA among IBP and physiological traits, and ion balance validation under salinity stress

**Fig. 4:**
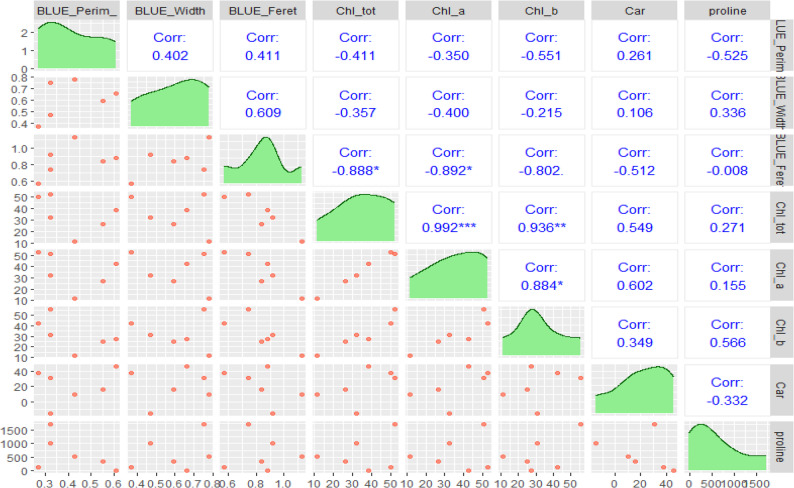
PCA analysis of variables and individuals related to the combination of IBP and physiological characteristics

**Fig. 5:**
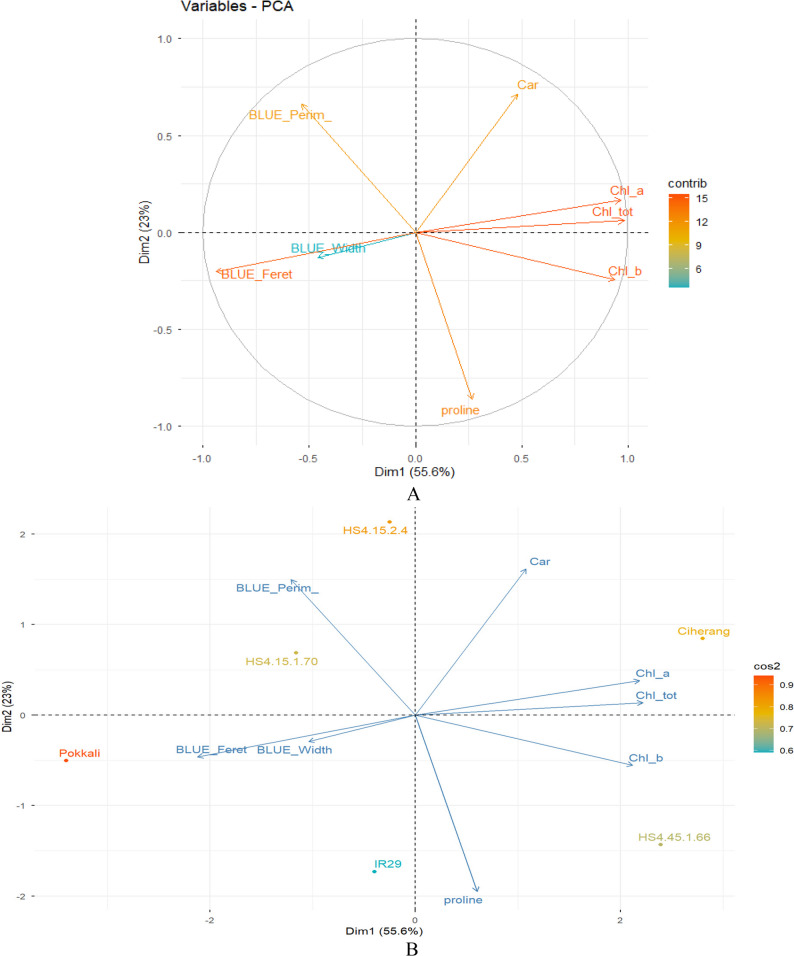
PCA analysis of variables contributions (**A**) and individuals clustering (**B**) related to the combination of IBP and physiological characteristics. Note: Dim: dimension, contrib = contribution, cos2 = representation quality of object, Chl_tot = chlorophyll total, Chl_a = chlorophyll a, Chl_b = chlorophyll b, Car= Carotenoid

The results of the correlation and PCA of the combined selected IBP characteristics and physiology are shown in Figs. 4 and 5, respectively. Based on Fig. 4, IBP Feret is significantly correlated with total chlorophyll (-0.888) and chlorophyll a (-0.892). Conversely, the IBP Width and Perim traits do not show significant correlations with other IBP and physiological traits. Nevertheless, IBP width tends to have a positive correlation with IBP Feret (0.609), and IBP Perim has a negative correlation with proline (-0.525).

Based on Fig. 5, the total diversity obtained from the two dimensions was 78.6% (Dim 1 = 55.6% and Dim 2 = 23%). The PCA analysis results showed that IBP width and Feret traits were inversely relationship with total chlorophyll, chlorophyll a, and chlorophyll b. Conversely, carotenoid physiological traits were independent and not associated with any IBP trait. Furthermore, based on the genotype grouping patterns, the Ciherang variety was grouped in quadrant (1) HS4.15.1.70 and HS4.15.2.4 were grouped in quadrant (2) The Pokkali and IR29 varieties were grouped in quadrant 3 at a considerable distance from the other varieties. The HS4.45.1.66 line was grouped in quadrant 4. Dim1 was mainly driven by IBP geometric traits, including BLUE_Feret, BLUE_MinFeret, BLUE_convex_Area, BLUE_Width, and BLUE_Perimeter, which showed strong positive loadings. In contrast, proline exhibited the opposite orientation relative to the IBP perimeter and width, indicating an inverse relationship between the osmotic response and plant geometric traits. Chlorophyll traits were also oriented opposite to width- and feret-related IBP traits, with no clear association with IBP traits. The genotype distribution revealed distinct grouping patterns in the PCA space. Pokkali was positioned in the positive region of Dim1, closely associated with geometric IBP traits. HS4.15.1.70 and HS4.15.2.4 clustered in the lower-right quadrant, while IR29 was located in the upper-left quadrant and Ciherang on the negative side of Dim1. HS4.45.1.66 was located in the lower-left quadrant.

**Fig. 6:**
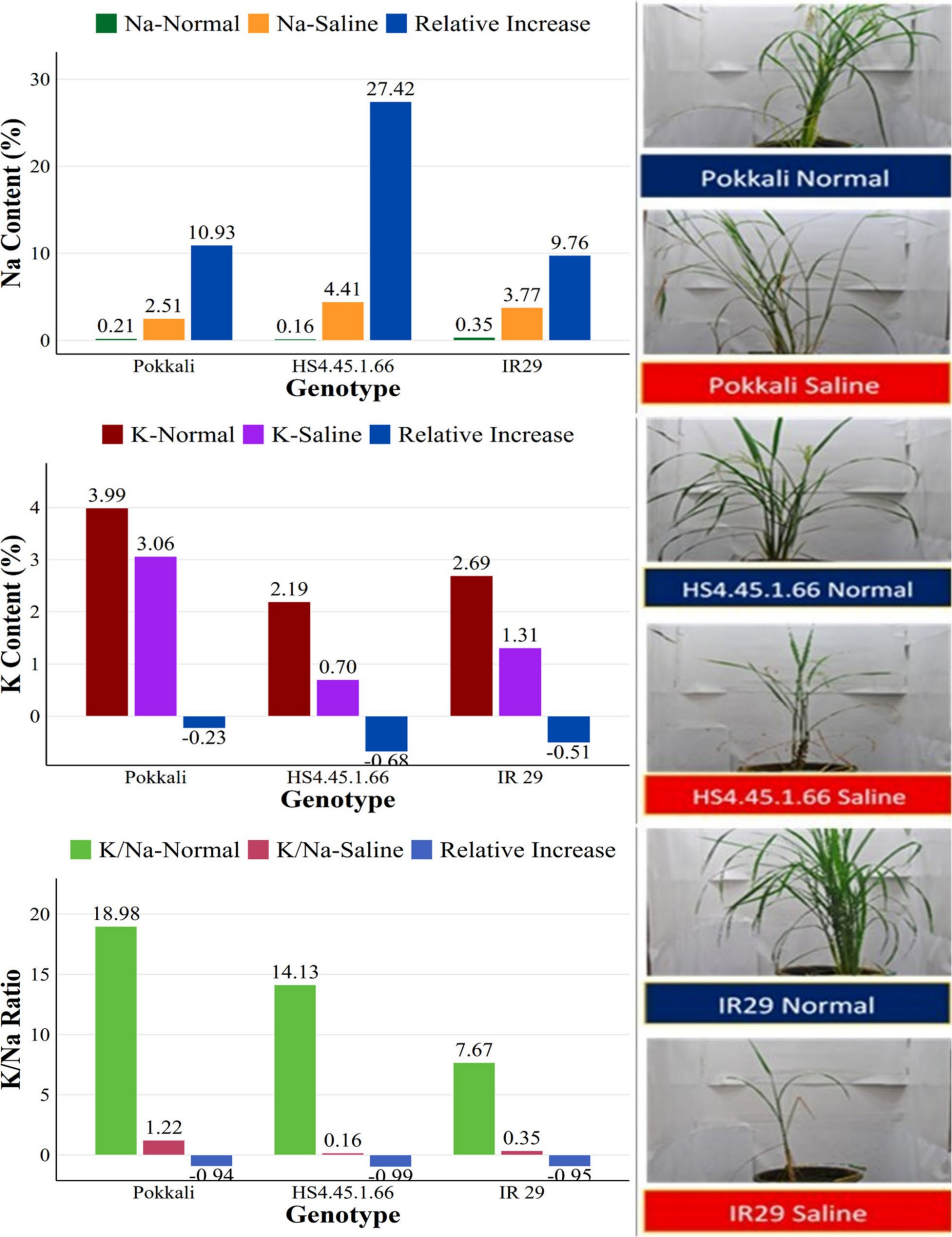
Comparative potassium and sodium contents in three representative genotypes of the tolerance group

The results of the analysis of Na^+^ and K^+^ content and the Na^+^/K^+^ ratio are shown in Fig. 6. Based on the potential Na ion content, all Pokkali, IR 29, and HS4.45.1.66 genotypes exhibited low Na ion content under normal conditions. This changed drastically when salinity stress was applied, increasing the Na^+^ ion content, particularly in the HS4.45.1.66 line (4.41). This was further reinforced by a high relative increase in that line (27.42). Based on K^+^ ion potential, all genotypes showed high K^+^ content under normal conditions, especially Pokkali, with a value of 3.99. Conversely, this K^+^ potential decreased under salinity conditions, with Pokkali remaining the variety with the highest K^+^ content (3.06) in the salinity treatment. This is further reinforced by the low relative increase in the Pokkali variety (-0.23). Based on the K^+^/Na^+^ content ratio, the Pokkali variety dominated under both normal and saline conditions. Conversely, the HS4.45.1.66 line had the lowest K^+^/Na^+^ ratio under saline conditions, and the IR29 variety had the lowest K^+^/Na^+^ ratio under normal conditions.

## Discussion

Environmental variations between normal and saline treatments resulted in non-significant genotype and genotype × environment (G×E) interactions for most IBP traits, indicating that environmental stress was the primary factor influencing phenotypic variation. Similar patterns have been documented [31], confirming that the evaluation criteria were based on the diversity between normal and saline environments. In general, evaluation in determining the level of stress tolerance requires a moderately stressful environment. This aims to create a more proportional diversity in the evaluation and selection process [25, 46, 54]. However, in this case it is still tolerable with considering the potential of the coefficient of variation (CV) and the effects of environmental diversity. Based on this, convex area, saturation area, perimeter, width, major, feret, minferet, and roundness emerged as potential IBP candidate characteristics for selection in the artificial screening of soil salinity in pots. The evaluation of genotypic responses to soil salinity stress under controlled conditions revealed that the mean Stress Tolerance Index (STI) of selected IBP traits remained below 1, indicating limited tolerance performance, including the Pokkali variety as an indicator of salinity stress tolerance in rice. These findings are consistent with previous study [25]. Generally, an STI value of 1 is considered the standard for genotype tolerance to salinity. This suggests that salinity stress was substantial, resulting in most genotypes being classified as moderately or highly sensitive [31, 67–69]. This underscores the importance of using the Best Linear Unbiased Estimator (BLUE) with STI to assess genotype tolerance to soil salinity stress in pots, as this combination can reflect the optimal adaptability potential of a genotype without confounding environmental effects [25, 70]. This analytical approach can serve as a foundation for analyzing IBP evaluation criteria for screening saline soils in pots. Through multivariate analysis using Principal Component Analysis (PCA) and factor analysis, perimeter, width, and Feret were identified as the most informative criteria for IBP selection.

The plant perimeter indicates morphological continuity, which is better preserved in salinity-tolerant genotypes. It quantifies object circumference length in images, reflecting complex shapes. Tolerant genotypes maintain growth, resulting in a higher perimeter than sensitive ones [25, 31, 67, 71–76]. Width represents the horizontal length (X-axis) of the minimum bounding rectangle. Rice tolerance correlates with tiller numbers; salinity-tolerant rice shows more tillers, influencing image width. Tolerant genotypes showed greater X-axis width than sensitive ones [25, 67, 71, 72, 77]. The Feret diameter denotes maximum distance between boundary points parallel to horizontal direction. Salinity stress reduces Feret potential due to osmotic stress and reduced growth; however, tolerant genotypes maintain stability, leading to higher Feret index [25, 31, 67, 73]. These characteristics show geometric potential differences between tolerant and sensitive genotypes. Tolerant genotypes sustain growth potential, correlating with enhanced geometric morphology. This is evident from STI Perimeter and Width, where Ciherang and IR29 showed low values. For Feret, Ciherang remained lowest, while IR29 showed higher values due to stress-induced leaf bending rather than tolerance mechanisms. Changes in leaf posture can alter geometric measurements, making IR29 appear similar to tolerant genotypes. The STI potential of IR29 feret under normal conditions is better than average, resulting in high IR29 feret potential, despite experiencing decline [25, 54, 67]. This is supported by physiological data that is sensitive. When combined with physiological indicators, these IBP traits effectively distinguished tolerant (Pokkali, HS4.15.1.70, and HS4.15.2.4), moderately tolerant (HS4.45.1.66), and sensitive (Ciherang and IR29) genotypes. The perimeter, width, and Feret serve as screening indicators under controlled conditions. Integration of physiological data through BLUE values, relative decline analysis, and PCA validated IBP-based discrimination and minimized posture-related misclassification. Differences in genotype clustering reflect variable influence, with physiological traits affecting genotype proximity in combined analysis, while structural traits dominate IBP-only PCA. Integration of physiological data through BLUE, relative decline analysis, and PCA has been reported by Anshori et al. [25]. This concept strengthens IBP evaluation criteria under hydroponic salinity screening and elucidates genotype responses to salinity stress. Therefore, integrating these physiological parameters can be applied in this study.

Based on the integration revealed, the perimeter exhibited a negative correlation with the relative proline increase, while the width and Feret demonstrated a negative correlation with the relative reduction in total chlorophyll, chlorophyll a, and b. Although, IBP Feret is considered to better represent the potential relationship to chlorophyll parameters. Moreover, IBP Width can also be represented by IBP Feret, making IBP Feret more suitable to be used as a criterion for evaluating the geometric IBP related to the chlorophyll components of rice leaves when experiencing salinity stress. The negative correlation in this case is positively oriented, meaning that the more adaptive one is, the smaller the relative decrease in chlorophyll, so negative correlation is highly regarded in this context. Feret correlation with higher salinity tolerance maintains greater chlorophyll stability and less pigment loss [78–80]. More tolerant plants show smaller decreases in chlorophyll, indicating better photosynthetic preservation in tolerant genotypes [25, 81–83]. IBP-derived width and Feret traits reflect photosynthetic stability under salinity stress with physiological indicators, serving as criteria for early-stage salinity screening, consistent with previous studies (Table 6). Perimeter characteristics showed a potential relationship with proline, indicating plant response to osmotic stress. Under salinity conditions, water remains available but cannot be absorbed due to elevated external solute concentrations [84–86]. Sensitive genotypes lacking effective tolerance mechanisms show excessive proline accumulation to maintain cellular water balance, indicating stress severity rather than adaptive tolerance. These genotypes experience accelerated physiological decline compared to tolerant ones, which maintain osmotic balance with lower proline levels [25, 87–89]. This contrast showed that perimeter-related IBP traits capture differences in stress response and can differentiate tolerant and sensitive genotypes when evaluated using physiological indicators. The perimeter, width, and Feret are complementary geometric indicators suitable for artificial pot-based salinity screening, supporting early-stage genotype differentiation under controlled conditions. Although IBP traits were primarily responsive to salinity stress, their ability to discriminate between genotypes should not be interpreted alone [25]. The stress-driven response of geometric traits reflects whole-rice plant adjustments under saline conditions, while genotype differentiation becomes more robust when IBP traits are evaluated using physiological indicators [25, 90–92]. IBP Perimeter and Feret traits are effective for choosing plants in pots to test for salt stress, showing stress effects that match plant tolerance, as seen in past studies. They support, rather than replace, other methods to distinguish plant types (Table 6).

**Table 6:** Image-based phenotyping traits (perimeter, width, and feret) as stress indicators in cereal crops

Reference	Country	Commodity & Population	Methods	Major Finding (Perimeter / Width / Feret Focus)
[71]	USA / Australia	Rice, 378 genotypes	Visible + fluorescence imaging, GWAS	Growth decline under salinity was captured through reductions in projected shoot width and perimeter, both genetically linked to Na⁺/K⁺ regulation on Chr 1 & 3.
[77]	South Korea, Pakistan	Rice (cv. Donggin)	Infrared imaging, physiology	Leaf width contraction correlated with temperature rise under salt stress; indicated reduced turgor linked to stress severity.
[72]	China	Rice, pot trials	RGB imaging, vertical structure analysis	Drought caused reduced canopy width and altered leaf perimeter curvature; both were reliable image-based stress indicators.
[67]	Indonesia	Rice, 8 genotypes	Fiji-based RGB imaging, PCA, STI	Perimeter and feret diameter emerged as main discriminators separating tolerant vs. sensitive genotypes under combined stress.
[31]	Indonesia	Rice, 8 genotypes	RGB imaging (Fiji 1.54c), STI, PCA	Perimeter, convex area, and feret length strongly correlated with tolerance indices; high feret values characterized drought–salinity-tolerant lines.
[25]	Indonesia	Rice, 10 DH lines	Hydroponic 0–120 mM NaCl, Fiji IBP, PCA, STI	Width, feret, and perimeter-based morphometric indices effectively ranked DH lines; these geometric traits aligned with field yield and ion balance.
[73]	Iran	Wheat, 3 cultivars	RGB imaging, 2D grain shape indices	Width (MinFeret) and perimeter-derived indices closely predicted grain weight loss under drought; high correlation (*r* ≥ 0.96) with TGW.

The findings of this study elucidate the mapping patterns and responses of each genotype to the interaction between IBP criteria and physiological factors, as analyzed both PCA biplots and Na^+^-K^+^ ion content. This interaction revealed that the Pokkali, HS4.15.1.70, and HS4.15.2.4 varieties, which are relatively tolerant, are distinctly separate from sensitive varieties such as IR 29 and Ciherang. This distinction also encompasses the moderate line HS4.45.1.66, identified as a moderate genotype [25, 54]. However, discrepancies arise in the results of the independent PCA biplot mapping of the IBP characteristics. Although IR29 is susceptible and clearly distanced from tolerant varieties, it clusters with the Pokkali variety in the PCA mapping of IBP-physiology interactions and is distinct from the Ciherang variety. Furthermore, the moderate line HS4.45.1.66 was an outlier in quadrant 4, positioned outside the range of tolerant and susceptible varieties. This underscores the complexity of the physiological response of strains to salinity stress. This phenomenon has also been documented by [25] and [54]. Generally, salinity stress exerts diverse effects on rice, leading to varied physiological and molecular response patterns and tolerance mechanisms among genotypes.

**Fig. 7:**
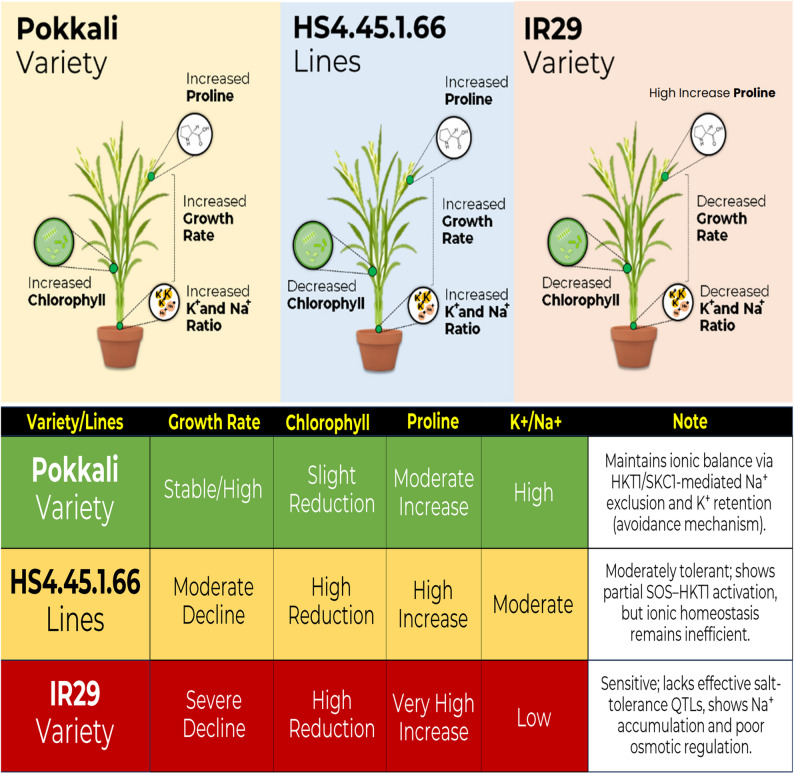
Morphophysiological and ionic responses of rice genotypes under salinity stress. Note: Pokkali Variety (Tolerant); HS4.45.1.66 (Moderate); IR29 (Senstive)

Genotypes HS4.15.1.70 and Pokkali showed similar physiological responses to salinity tolerance, maintaining stable chlorophyll and proline levels. This is supported by the elevated K⁺/Na⁺ ratio in Pokkali, suggesting tolerance through avoidance. This tolerance contrasts with HS4.15.2.4, which showed decreased chlorophyll and minimal proline increase, indicating tolerance based on growth rate ratio and compensation of salinity stress effects. HS4.45.1.66 applies a similar concept but shows a small growth-to-impact ratio, demonstrated by high chlorophyll decrease, proline increase, elevated Na content, and a K⁺/Na⁺ ratio superior to IR29 under saline conditions. This indicates a different pattern, placing this genotype as an outlier in quadrant 4, apart from both the tolerant and sensitive groups. This genotype strives to enhance its growth as part of a tolerance or avoidance pattern in response to salinity stress, so phenotypically it is relatively superior compared to sensitive genotypes. However, physiologically, this genotype is heavily impacted in its efforts to maintain growth under salinity stress. This makes the HS4.45.1.66 genotype classified as a “moderately tolerant genotype”. In contrast, IR29 and Ciherang, categorized as sensitive, showed distinct responses. IR29 maintained chlorophyll decline with high proline increase but showed a low K⁺/Na⁺ ratio, indicating it mitigates stress by reducing growth rate. The ratio between growth rate and impact is minimal, with no genetic potential supporting tolerance, causing mortality under saline conditions. Ciherang failed to maintain growth rate and chlorophyll potential under high salinity, leading to sensitivity to insufficient photosynthate production (Fig. 7 and Table 7). This integrative classification reflects the multidimensional nature of salt tolerance, where genotypes may rely on different mechanisms (avoidance vs. tolerance) and display trait-dependent variation. Therefore, comprehensive tolerance testing, including physiological assessments, is essential for salinity screening, including artificial soil screening in pots.

**Table 7:** Comparative physiological responses of rice genotypes to salinity stress

Tolerance	Growth Rate	Chlorophyll Pigments	K^+^/Na^+^ ratio	Proline Content	Physiological Description	Reference
Tolerant (Pokkali)	Stable, slightly decreasing	Sustained (high SPAD, stable Fv/Fm)	High (> 2.0)	High (Adaptive)	Active adaptation to stress; ion selectivity and osmoregulation	[62, 99, 100]
Moderate	Moderate decline	Partially decreased	Moderate (1.0–1.5)	High (Compensation)	Mixed response; partly adaptive, mainly stress-induced	[32, 39, 50]
Sensitive (IR29)	Decreased significantly	Rapidly declining	Low (< 0.5)	High (Reactive)	Stress response; proline increases as a sign of cell damage	[21, 50, 101]

Molecular mechanisms, particularly those associated with ionic homeostasis, osmotic adjustment, and oxidative detoxification. Notably, these adaptive mechanisms enable tolerant genotypes to sustain functional growth under salinity stress, rather than merely diminishing growth as a result of stress injury. Tolerant varieties, such as Pokkali, demonstrate a more coordinated molecular response through the robust activation of ion homeostasis-regulating genes, including OsHKT1;5 (SKC1), which facilitates the withdrawal of Na⁺ ions from the xylem to the roots, thereby preventing their accumulation in the photosynthetic tissues. Furthermore, the expression of OsSOS1 and OsNHX1 is significantly elevated, functioning in the exclusion of Na+ from the plasma membrane and the sequestration of Na+ into the vacuole [93, 94]. The activation of K⁺ transporters, including OsHAK and OsAKT1, aids in maintaining a high K⁺/Na⁺ ratio, thus supporting enzymatic stability and photosynthesis (Table 8).

**Table 8:** Main biomolecular mechanisms determining salinity tolerance in rice

Category Mechanism	Gene / Protein / Molecule	Verified Molecular Function	Role in Tolerance vs. Sensitivity	Reference
Ionic Homeostasis (Na+/K+)	*OsHKT1;5 (SKC1)*	Na+ transport from xylem → reduces Na + in leaves	High expression = tolerant; low = sensitive	[63, 105]
Ionic Homeostasis	*OsSOS1*	Na+/H+ antiporter → removes Na+ from cells	Tolerance removes Na+ more efficiently	[106, 107]
Ionic Homeostasis	*OsNHX1*	Na+ sequestration into vacuoles	Tolerance stores Na+ safely; sensitivity to Na+ damages the cytosol	[63]
K+ Transport	*OsHAK / OsAKT1*	Maintains stable K+ levels	High K+/Na+ ratio = tolerant	[93, 94]
Osmotic Adjustment	*Proline / P5CS*	Proline biosynthesis for osmoregulation	Tolerance = adaptive proline; sensitivity = reactive/damage	[95]
Osmotic Adjustment	*Trehalose*,* Sucrose*	Stabilization of cell structure and turgor	High sugar tolerance; decreased sensitivity	[95, 96]
ROS Detoxification	*SOD*,* CAT*,* APX*	Removes ROS to prevent membrane and chlorophyll damage	Low ROS tolerance; high ROS sensitivity	[97, 98]
Cell Protection	*LEA / Dehydrin*	Protects membranes and proteins from dehydration and toxic ions	Tolerance increases during stress; sensitivity is not optimal	[108, 109]
Cell Protection	*HSP70/HSP90*	Anti-protein misfolding chaperones	Tolerance is stable; sensitivity is impaired	[110]
Gene Regulation	*DREB/CBF*	Activation of adaptive genes (ions, osmolytes, antioxidants)	Tolerance is strongly expressed; sensitivity is weak	[111, 112]
Gene Regulation	*NAC TFs*	Regulation of ROS, senescence, and apoptosis	Tolerance is protective; sensitivity cell death	[102, 103]
Gene Regulation	*bZIP TFs*	Regulation of ROS and hormone pathways	Tolerance maintains homeostasis; sensitivity to ROS dysfunction	[113]
Signaling	*MAPK / SAPK4*	Activation of salinity stress response pathways	Overexpress SAPK4 → increased tolerance	[114, 115]
Tolerance QTL	*Saltol (OsHKT1;5)*	Control of K+/Na+ ratio (major QTL for salinity tolerance)	Pokkali carries the Saltol allele, whereas IR29 does not	[56]

Salinity tolerance in rice is attributed to the integration of several critical physiological and Osmotic adjustment mechanisms are also more efficient in tolerant varieties, characterized by increased accumulation of proline controlled by *P5CS*, as well as compatible sugars such as trehalose and sucrose [95, 96], which preserve the basic structure of membranes and proteins. Concurrently, the antioxidant system, including *SOD*,* CAT*, and *APX*, consistently increases, effectively suppressing ROS accumulation and protecting chloroplasts, cell membranes, and other organelles from oxidative damage [97, 98]. Protective proteins, such as *LEAs*,* dehydrins*, and *HSPs*, are strongly induced, further enhancing cellular stability under extreme ionic conditions. These coordinated responses reflect adaptive tolerance mechanisms rather than stress-induced growth inhibition.

In contrast, sensitive varieties like IR29 show weaker activation of these molecular pathways. Na⁺ ions accumulate in the leaves, leading to decreased K⁺/Na⁺ ratio and increased ROS production that damages membranes, proteins, and chlorophyll. These reductions in plant geometry indicate physiological injuries and impaired cellular functions rather than adaptive tolerance. The activation of key transcription factors *DREB/CBF*,* NAC*, and *bZIP*, which regulate stress response genes, is also lower than in tolerant varieties [102–104]. Moderately tolerant varieties, such as HS4.45.1.66, showed a transitional response with increased activation of ion transporters, osmolytes, antioxidants, and protective proteins, though not as robust as Pokkali. Genetically, the Saltol QTL containing OsHKT1; 5 serves as the primary differentiator among tolerance groups. Thus, variations in ion balance regulation, osmotic stability, oxidative stress suppression, and stress-responsive gene networks account for the observed gradient of salinity tolerance among tolerant, moderate, and sensitive rice genotypes.

## Conclusion

This study demonstrates that the integration of image-based phenotyping (IBP) with physiological analysis provides a practical and reliable framework for salinity screening in rice under controlled pot conditions in the greenhouse. Perimeter and Feret traits were identified as supportive image-based indicators, whose relevance was validated when interpreted together with physiological responses, as shown by BLUE analysis, relative reduction metrics, and exploratory PCA. Rather than serving as standalone classifiers, these geometric traits capture stress-responsive morphological patterns linked to osmotic regulation and to photosynthetic stability. PCA-based grouping further indicated differential genotype responses to artificial salinity stress, ranging from tolerant to moderate and sensitive; however, such grouping is context-dependent and influenced by the trait sets included. Therefore, a detailed physiological evaluation is essential to distinguish adaptive tolerance mechanisms from stress-induced growth inhibition and posture-related effects, thereby improving the accuracy of genotype phenotyping under salinity stress. Nevertheless, these IBP criteria need to be tested on a larger genotype to elevate their status from evaluation criteria to selection criteria. Furthermore, the integration with molecular and multi-omics approaches will be valuable for further elucidating genotype-specific tolerance mechanisms and strengthening the translational relevance of IBP-assisted selection in rice breeding programs.

## Supplementary Information

Supplementary Material 1

Supplementary Material 2

## Data Availability

The data presented in this study are available upon request.

## Abbreviations

BLUE: Best Linear Unbiased Estimator
Sat_Area: Saturation area
Perim: Perimeter
MinFeret: Minimum of ferret
Round: Roundness
