# Supplementary material for: Geometric image-based phenotyping and physiological analysis for validation of rice salinity tolerance screening under artificial pot conditions
Source: BMC Plant Biol. 2026 Apr 23;26:947. doi: 10.1186/s12870-026-08810-5 (PMC13227774; doi:10.1186/s12870-026-08810-5)
Supplement: Supplementary file 1 — Supplementary Material 1 [file 12870_2026_8810_MOESM1_ESM.docx]

**Supplementary 1**. The Best linear unbiased estimator (BLUE) analysis of IBP characters in the salinity screening method in pots

| Environment | Genotype | convex_Area | Gren_Area | Sat_Area | sat_conv | Perim_ | Width | Major | Minor | Feret | IntDen | FeretX | FeretY | MinFeret | AR | Round |
| --- | --- | --- | --- | --- | --- | --- | --- | --- | --- | --- | --- | --- | --- | --- | --- | --- |
| normal | HS4.15.1.70 | 2087.33 | 705.23 | 744.87 | 0.36 | 1756.00 | 48.68 | 49.44 | 18.46 | 72.99 | 44346.67 | 1625.67 | 864.33 | 45.27 | 2.80 | 0.38 |
| normal | HS4.15.2.4 | 2343.33 | 676.40 | 733.40 | 0.32 | 2102.67 | 50.19 | 45.39 | 19.93 | 74.85 | 46586.67 | 1483.00 | 1690.00 | 45.49 | 2.48 | 0.46 |
| normal | HS4.45.1.66 | 1882.37 | 444.93 | 1343.33 | 1.19 | 1415.23 | 59.32 | 47.65 | 31.63 | 72.08 | 159530.00 | 1203.33 | 556.00 | 50.05 | 1.69 | 0.65 |
| normal | Ciherang | 1556.00 | 487.36 | 1649.93 | 1.38 | 1203.57 | 51.38 | 57.30 | 32.38 | 73.66 | 199746.67 | 1228.00 | 1697.00 | 48.90 | 2.15 | 0.52 |
| normal | IR29 | 2569.33 | 545.71 | 873.97 | 0.34 | 1489.33 | 46.07 | 58.70 | 35.07 | 83.74 | 141340.00 | 1277.15 | 1549.00 | 46.58 | 2.51 | 0.45 |
| normal | Pokkali | 2728.00 | 402.61 | 739.00 | 0.35 | 1613.00 | 57.67 | 50.94 | 30.19 | 83.79 | 99383.33 | 789.96 | 1681.00 | 47.45 | 2.50 | 0.44 |
| Average | | 2194.39 | 543.71 | 1014.08 | 0.66 | 1596.63 | 52.22 | 51.57 | 27.94 | 76.85 | 115155.56 | 1267.85 | 1339.56 | 47.29 | 2.36 | 0.48 |
| Saline | HS4.15.1.70 | 1213.00 | 195.22 | 201.04 | 0.16 | 794.83 | 33.13 | 31.24 | 7.77 | 67.71 | 10534.33 | 1971.67 | 1768.33 | 27.89 | 3.89 | 0.27 |
| Saline | HS4.15.2.4 | 1304.00 | 189.32 | 273.80 | 0.20 | 742.93 | 35.95 | 41.58 | 17.68 | 69.56 | 77220.00 | 1933.33 | 2527.33 | 30.16 | 3.14 | 0.39 |
| Saline | HS4.45.1.66 | 1092.50 | 107.15 | 142.57 | 0.13 | 571.03 | 34.68 | 38.82 | 15.43 | 60.56 | 77752.00 | 1935.00 | 2461.00 | 16.95 | 3.15 | 0.49 |
| Saline | Ciherang | 926.73 | 159.34 | 247.17 | 0.15 | 568.98 | 19.83 | 29.56 | 9.39 | 45.62 | 24238.33 | 1661.33 | 1089.33 | 16.10 | 4.81 | 0.29 |
| Saline | IR29 | 1021.20 | 142.15 | 407.23 | 0.12 | 548.83 | 27.68 | 42.66 | 10.35 | 64.66 | 48148.33 | 2038.00 | 2537.00 | 24.50 | 4.28 | 0.24 |
| Saline | Pokkali | 1740.97 | 164.55 | 473.19 | 0.10 | 676.43 | 36.89 | 45.39 | 16.36 | 79.93 | 83361.67 | 1988.00 | 1921.33 | 29.83 | 4.88 | 0.32 |
| Average | | 1216.40 | 159.62 | 290.84 | 0.15 | 650.51 | 31.36 | 38.21 | 12.83 | 64.68 | 53542.44 | 1921.22 | 2050.72 | 24.24 | 4.03 | 0.33 |
