# Supplementary material for: Geometric image-based phenotyping and physiological analysis for validation of rice salinity tolerance screening under artificial pot conditions
Source: BMC Plant Biol. 2026 Apr 23;26:947. doi: 10.1186/s12870-026-08810-5 (PMC13227774; doi:10.1186/s12870-026-08810-5)
Supplement: Supplementary file 2 — Supplementary Material 2 [file 12870_2026_8810_MOESM2_ESM.docx]

**Supplementary 2**. The Results of the Best linear unbiased estimator (BLUE) analysis of Physiological traits in the salinity screening method in pots

| Environment | Genotype | BLUE_total (μg g¯¹ FW) | BLUE_Ca (μg g¯¹ FW) | BLUE_Cb (μg g¯¹ FW) | BLUE_Car | BLUE_prolin (μmol g¯¹ FW) | Na (%) | K (%) |
| --- | --- | --- | --- | --- | --- | --- | --- | --- |
| Normal | HS4.15.1.70 | 45.73 | 33.75 | 11.98 | 9.09 | 2.83 | NS | NS |
| Normal | HS4.15.2.4 | 58.96 | 43.41 | 15.55 | 11.45 | 3.47 | NS | NS |
| Normal | HS4.45.1.66 | 57.07 | 39.47 | 17.60 | 11.47 | 4.32 | 0.16 | 2.19 |
| Normal | Ciherang | 48.05 | 35.40 | 12.65 | 8.71 | 4.52 | NS | NS |
| Normal | IR29 | 45.38 | 33.27 | 12.11 | 8.15 | 2.27 | 0.35 | 2.69 |
| Normal | Pokkali | 44.64 | 32.80 | 11.83 | 8.95 | 1.98 | 0.21 | 3.99 |
| Average | | 49.97 | 36.35 | 13.62 | 9.64 | 3.23 | 0.24 | 2.95 |
| Saline | HS4.15.1.70 | 33.68 | 24.72 | 8.96 | 7.68 | 11.96 | NS | NS |
| Saline | HS4.15.2.4 | 36.21 | 24.88 | 11.34 | 6.18 | 3.14 | NS | NS |
| Saline | HS4.45.1.66 | 27.18 | 19.38 | 7.80 | 7.92 | 78.83 | 4.41 | 0.70 |
| Saline | Ciherang | 23.97 | 16.67 | 7.30 | 5.40 | 10.57 | NS | NS |
| Saline | IR29 | 30.93 | 22.55 | 8.37 | 9.39 | 24.96 | 3.77 | 2.69 |
| Saline | Pokkali | 39.50 | 29.00 | 10.50 | 8.05 | 12.17 | 2.51 | 3.06 |
| Average | | 31.91 | 22.86 | 9.04 | 7.44 | 23.61 | 3.56 | 2.15 |

Note : NS: no Sample
